# Sex-specific associations of SHBG, testosterone, and obesity with cholecystectomy risk: a population-based cohort study

**DOI:** 10.1186/s13293-026-00884-y

**Published:** 2026-03-23

**Authors:** Jie-Qiong Lyu, Yi Luo, Yu-Wen Qian, Ji-Mei Gu, Meng-Yuan Miao, Guo-Chong Chen, Guo-Jian Yin

**Affiliations:** 1 The Fourth Affiliated Hospital (Medical Center of Soochow University), School of Public Health, Suzhou Medical College of Soochow University, Suzhou, China; 2 Department of Plastic Surgery, Dermatology Hospital of Southern Medical University, Guangzhou, China

**Keywords:** Cholecystectomy, Gallstone disease, Obesity, Sex hormone-binding globulin, Testosterone

## Abstract

**Background:**

Gallstone disease exhibits a marked sex difference. While obesity and female sex hormones are known risk factors, the role of sex hormone-binding globulin (SHBG) and testosterone in gallstone-related risk, and whether they mediate the association between obesity and gallbladder disease, remain unclear.

**Methods:**

We included 176,909 men and 160,147 women from the UK Biobank. Serum SHBG and testosterone were measured using immunoassays. Incident cholecystectomy cases were identified through hospital records. Multivariable Cox proportional hazards models were used to calculate hazard ratios (HRs) with 95% confidence intervals (CIs). Mediation analyses were performed to assess the contribution of SHBG and testosterone to the obesity-cholecystectomy association.

**Results:**

A total of 2,877 men and 4,607 women underwent cholecystectomy. Higher SHBG levels were associated with a lower risk of cholecystectomy in both sexes, with a stronger association in women (HR_Q4 vs. Q1_ = 0.40, 95% CI: 0.36–0.43) than in men (HR_Q4 vs. Q1_ = 0.67, 95% CI: 0.59–0.77). Elevated testosterone levels were associated with an increased risk of cholecystectomy only in women (HR_Q4 vs. Q1_ = 1.28; 95% CI: 1.18–1.38). Obesity exhibited a stronger association with cholecystectomy in women than in men. In women, SHBG mediated 14.7% and 20.0% of the associations between general and abdominal obesity and cholecystectomy, respectively, whereas testosterone mediated smaller proportions (2.7% and 1.8%). No significant mediation was observed in men.

**Conclusions:**

SHBG levels are inversely associated with risk of cholecystectomy in both sexes, whereas higher testosterone levels are associated with a higher risk of cholecystectomy only in women. SHBG and, to a lesser extent, testosterone accounted for a modest proportion of the obesity-cholecystectomy association in women.

**Supplementary Information:**

The online version contains supplementary material available at 10.1186/s13293-026-00884-y.

## Highlights

Higher levels of SHBG are related to a reduced risk of cholecystectomy in both sexes.

Elevated testosterone levels are associated with an increased risk of cholecystectomy in women but not in men.

Obesity shows a stronger association with cholecystectomy in women than in men.

SHBG and, to a lesser extent, testosterone may account for a modest proportion of the obesity-cholecystectomy association in women.

**Supplementary Information:**

The online version contains supplementary material available at 10.1186/s13293-026-00884-y.

## Key summary for a broad audience

Gallstones and gallbladder removal (also called cholecystectomy) are much more common in women than in men. Although obesity and female sex hormones are known risk factors, it has been unclear whether sex hormone-binding globulin (SHBG) and testosterone also play a role, and whether they help explain the higher risk seen in women and people with obesity.

We studied over 330,000 adults in the UK who were free of gallbladder disease at the start of the study. All participants had their blood levels of SHBG and testosterone measured, and their health was tracked over time using hospital records to identify those who later underwent gallbladder removal.

We found that people with higher SHBG levels had a lower chance of needing gallbladder surgery. This pattern was seen in both sexes but was evidently stronger in women. Testosterone showed a different pattern: higher testosterone levels were linked to a greater risk of gallbladder removal in women, but not in men. We also discovered that in women, part of the reason obesity increases the chance of gallbladder surgery is that obesity affects these hormone levels, especially SHBG. In men, however, these hormones did not explain the link between obesity and gallbladder problems.

Overall, our findings provide new insight into how hormones may link obesity to gallbladder problems. More research is needed to better understand the biological processes behind these findings.

**Supplementary Information:**

The online version contains supplementary material available at 10.1186/s13293-026-00884-y.

## Introduction

Gallstone disease is highly prevalent in developed countries, with an occurrence rate ranging from 10% to 15% in the US [1] and approximately 15% in the UK [2]. Although most gallstones remain asymptomatic, up to 20% of patients develop complications such as cholecystitis, pancreatitis, or biliary obstruction [3], often requiring cholecystectomy, the surgical removal of the gallbladder [4]. As one of the most frequently performed abdominal surgeries, cholecystectomy imposes a substantial burden on healthcare systems, contributing significantly to medical costs, hospital admissions, and patient morbidity [5, 6].

A consistent sex difference exists in gallstone disease: women have a considerably higher risk than men, accounting for roughly 70% of all cholecystectomies [7, 8]. This difference points to a potential role of sex hormones in the pathophysiology of gallstone formation. Population-based studies have shown that increased estrogen exposure, including pregnancy and postmenopausal hormone replacement therapy (HRT), is associated with a higher risk of gallstone disease [9–11]. Randomized controlled trials have also demonstrated that HRT increases the risk of gallstone-related complications in postmenopausal women [12]. Experimental studies indicate that estrogen receptor-mediated signaling enhances hepatic cholesterol secretion and increases cholesterol saturation of bile [13–15], while progesterone impairs gallbladder smooth muscle contractility and delays gallbladder emptying [16, 17]. In contrast, the role of androgens, particularly testosterone, in gallbladder disease remains less clearly defined. While androgens are known to influence hepatic lipid metabolism and insulin sensitivity [18], population-based evidence linking circulating testosterone to gallstone disease remains scarce [19, 20], underscoring the need for further investigation of testosterone and its binding protein, sex hormone-binding globulin (SHBG), in gallstone-related outcomes. SHBG is synthesized primarily in hepatocytes and closely reflects hepatic insulin resistance and lipid metabolism—processes central to cholesterol supersaturation of bile [21, 22]. Testosterone exhibits marked sex-specific regulation and is strongly influenced by adiposity and metabolic status, both of which are established risk factors for gallstone disease [23–25]. Clarifying the roles of SHBG and testosterone may therefore provide insight into hormonal and metabolic pathways underlying sex differences in gallstone-related complications.

As a well-established risk factor for gallstone disease, obesity is known to influence circulating sex hormone levels. Some evidence suggests that the association between obesity and gallstone disease is stronger in women than in men [26]. Prior research has also suggested that sex hormones may partially mediate the effects of obesity on sex-specific health outcomes [27, 28]. Nevertheless, it remains unclear whether SHBG and testosterone mediate the observed association between obesity and the risk of cholecystectomy, particularly in a sex-specific manner. Clarifying these interrelationships is critical for understanding the biological mechanisms that drive sex differences in gallbladder disease. Such insights could help identify hormonal biomarkers and inform sex-specific strategies for prevention, particularly in metabolically at-risk women.

To address these gaps, we conducted a large-scale prospective study using data from the UK Biobank to examine (1) the sex-specific associations of serum SHBG and testosterone levels with the risk of cholecystectomy and (2) the extent to which SHBG and testosterone mediate the relationship between obesity and cholecystectomy in men and women. By integrating sex-stratified analyses with formal mediation models in a large population-based cohort, this study extends current understanding of hormonal pathways linking obesity to gallstone-related complications.

## Methods

### Study design and population

The UK Biobank is a large prospective cohort study that aims to investigate the primary causes of various chronic diseases, including genetic, environmental, and lifestyle factors [29]. Details of the study protocol are available online (https://www.ukbiobank.ac.uk/media/gnkeyh2q/study-rationale.pdf). In brief, approximately 500,000 men and women aged 37 to 73 years were recruited between 2006 and 2010. Participants were invited to attend one of the 22 assessment centers located throughout England, Wales, and Scotland where they underwent questionnaire surveys, brief interviews, and physical measurements. The UK Biobank study was approved by the research ethics committee (REC reference for UK Biobank 11/NW/0382) and all participants provided informed consent at recruitment. All data collection and research were conducted in accordance with the Declaration of Helsinki.

Participants with a history of cholecystectomy (*n* = 8851), disorders of gallbladder, biliary tract, or pancreas (*n* = 10,709), cancer (except non-melanoma skin cancer; *n* = 43,562) at recruitment were excluded. We also excluded individuals who developed cancer in specific organs (the duodenum, liver, gallbladder, biliary ducts, or pancreas) (*n* = 1,968) or received liver transplantation (*n* = 48) during follow-up. Further excluded were participants without measurements of SHBG or testosterone (*n* = 96,958) and those who had the top 0.5% SHBG or testosterone levels in men or in women (*n* = 3,259). Consequently, a total of 337,056 individuals (176,909 men and 160,147 women) were included for analysis (Supplementary Fig. 1). This work has been reported in line with the STROCSS criteria [30].

### Blood sample collection and laboratory assays

During recruitment, venous blood samples were collected from all participants at assessment centers and stored at − 80 °C. Samples were collected at various times throughout the day according to when participants attended the assessment center. Samples were obtained under non-fasting conditions, with the time since the last food or drink intake recorded. Serum SHBG levels were measured using a two-step sandwich chemiluminescent immunoassay (DXI 800, Beckman Coulter, UK). Adult reference intervals for serum SHBG have been reported as approximately 12.6–92.4 nmol/L in men and 18.4-211.5 nmol/L in women [31]. Serum testosterone was assayed using a competitive binding chemiluminescent immunoassay (DXI 800, Beckman Coulter, UK). Comprehensive information regarding the assay techniques and quality assurance procedures can be accessed online (https://biobank.ndph.ox.ac.uk/showcase/showcase/docs/serum_biochemistry.pdf).

### Outcome ascertainment

The outcome of interest in this study was the occurrence of cholecystectomy during the follow-up period. We chose cholecystectomy as the primary outcome because it reflects clinically significant gallstone disease requiring medical intervention, whereas asymptomatic gallstones are often managed conservatively without routine treatment [32]. The procedures of cholecystectomy were identified using hospital inpatient records obtained from the Hospital Episode Statistics for England, Scottish Morbidity Record data for Scotland, and the Patient Episode Database for Wales. Incident cases of cholecystectomy and the corresponding date of the procedures were identified using UK Biobank field IDs 41,200 (operative procedures-main OPCS4) and 41,260 (date of first operative procedure-main OPCS4).

### Assessment of covariates

Selection of confounders was based on prior knowledge [33–35]. Information on sociodemographic factors, reproductive and medical histories, lifestyle behaviors, and medication use was collected at baseline by touchscreen questionnaires and nurse-led interviews. The Townsend deprivation index was derived by combining four census variables (unemployment, non-car ownership, non-home ownership, and household overcrowding). Measurements of blood pressure were performed using the Omron HEM-7015IT digital blood pressure monitor and calculated as the mean of two sitting measures. Physical activity was assessed at baseline using the self-reported short-form International Physical Activity Questionnaire and the data were summarized and reported in metabolic equivalent of task hours per week (MET-h/week). Several anthropometric measures were collected and body mass index (BMI) was calculated based on measured weight and height (kg/m²). General obesity was defined as BMI ≥ 30 kg/m^2^ and abdominal obesity was defined as waist circumference ≥ 102 cm for men and ≥ 88 cm for women. A variety of blood analytes (e.g., glycemic, lipid, and inflammatory biomarkers) were quantified by standard procedures using the blood samples collected at baseline.

### Statistical analysis

Baseline characteristics of the participants were described by sex-specific quartiles of SHBG for men and women separately. Continuous variables were presented as medians (interquartile range) and categorical variables as percentage. Spearman correlations were calculated among SHBG, testosterone, and a variety of other risk factors including age, BMI, waist circumference, glycosylated hemoglobin (HbA1c), total cholesterol, systolic blood pressure (SBP), and C-reactive protein (CRP).

Cox proportional hazards models were employed to estimate hazard ratios (HRs) and 95% confidence intervals (CIs) of cholecystectomy across the sex-specific quartiles of SHBG or testosterone, using the lowest quartile as the reference. The proportional hazards assumption was assessed using Schoenfeld residuals and no meaningful violations were detected. We also modelled SHBG and testosterone levels as continuous variables and estimated HRs (95% CI) of cholecystectomy for each additional 1-standard deviation (SD) increment in their levels. Person-time of follow-up was calculated as the duration from date of baseline evaluation until the date of performing cholecystectomy, date of death or withdrawal from the study, or date of the most recent follow-up, whichever occurred first. Three Cox models were constructed to incrementally adjust for potential confounders. Model 1 was adjusted for age (y), ethnicity (White, Asian or Asian British, Black or Black British, mixed ethnicities), and the Townsend deprivation index. Model 2 was adjusted for the covariates in model 1 and was further adjusted for smoking status (never, former, current), drinking status (never, former, current), total physical activity (MET-h/week), and for women, menopausal status (yes, no), and HRT (yes, no). The full model (model 3) was adjusted for all the above-listed covariates and was further adjusted for testosterone (for the analysis on SHBG) or SHBG (for the analysis on testosterone). Potential nonlinear relationships of SHBG, testosterone, and adiposity measures (i.e., BMI or waist circumference) with risk of cholecystectomy were examined using restricted cubic splines with four knots at the 5th, 35th, 65th, and 95th percentiles of the exposure distribution in men or women. Subgroup analyses were performed to examine the relationships between SHBG or testosterone levels and cholecystectomy risk in men and in women, according to the following participant characteristics: age, ethnicity, smoking status, drinking status, in addition to menopausal status and HRT use in women. We performed a series of supplementary and sensitivity analyses to assess the robustness of our findings. First, we excluded participants who had reproductive system or thyroid disorders which may affect hormone levels. Second, we excluded participants with cholecystectomies who were not diagnosed with cholelithiasis before the surgery. Incident cases of cholelithiasis and the corresponding date were identified using UK Biobank field ID 131,674 (Date K80 first reported). Third, we additionally adjusted the examined associations for certain dietary habits (i.e., coffee and tea consumption), metabolic factors (i.e., those reflecting inflammation, glucose or lipid metabolism, or blood pressure), or adiposity (i.e., BMI and waist circumference) which may affect SHBG or testosterone levels and the risk of cholecystectomy. Finally, we conducted a sensitivity analysis using calculated free testosterone (cFT), derived with the Vermeulen mass action equation based on measured total testosterone, sex hormone-binding globulin, and serum albumin [36, 37]. Sex-specific quartiles of cFT and per-standard deviation increments were modeled in sex-stratified Cox proportional hazards models.

We also examined the associations of BMI, waist circumference, and general or abdominal obesity status with risk of cholecystectomy after the multivariable adjustment (model 3). To estimate the proportion of the obesity-cholecystectomy association that could be mediated by SHBG or testosterone, we conducted mediation analyses using PROC CAUSALMED in SAS. These analyses were performed under the assumptions of no unmeasured confounding of the obesity-hormone and hormone-cholecystectomy relationships, conditional on the covariates included in the models. The analyses further assumed a temporal ordering in which obesity influences circulating hormone levels, which subsequently affect the risk of cholecystectomy. The statistical tests conducted in this study were two-sided and the analyses were performed using Stata software (version 15.1; StataCorp) and SAS (version 9.4).

## Results

### Baseline participant characteristics

Median levels of SHBG were 38.88 nmol/L in men and 61.30 nmol/L in women, and median levels of testosterone were 11.92 nmol/L in men and 1.10 nmol/L in women; these values are consistent with established reference ranges for adult populations. Supplementary Table 1 presents sex-specific characteristics of the study participants according to quartiles of SHBG levels in men and women. Regardless of sex, participants with higher levels of SHBG were more likely to be current smokers, had higher levels of physical activity, were less likely to report diabetes, hypertension, or hyperlipidemia, and had lower levels of BMI, waist circumference, HbA1c, and CRP. In men, higher SHBG levels were also associated with older age and lower likelihood of using non-steroidal anti-inflammatory drugs. In women, higher SHBG levels were also associated with younger age, lower levels of SBP, and a lower likelihood of using aspirin or HRT or being menopausal.

### SHBG and risk of cholecystectomy

During a median follow-up of 12.27 years in men and 12.39 years in women, 2877 and 4607 incident cases of cholecystectomy were recorded in men and women, respectively.

**Fig. 1:**
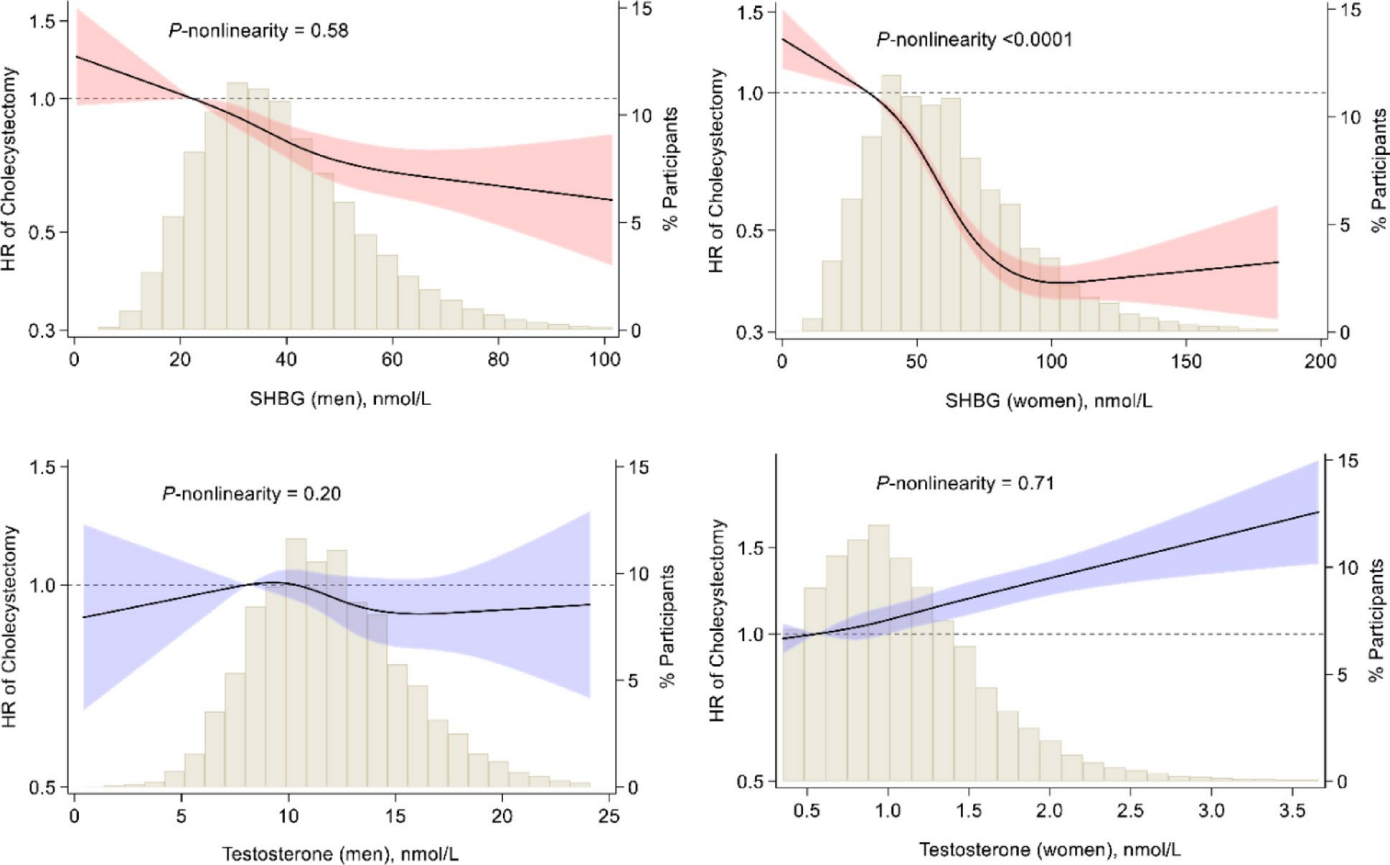
Dose-response relationships of serum SHBG and testosterone with risk of cholecystectomy in men and women. Median for the first quartile of sex hormone-binding globulin (SHBG) or testosterone was used as the reference. Results were adjusted for covariates listed for model 3 in Table 1 (for SHBG) or Table 2 (for testosterone)

After adjusting for sociodemographic factors, there were significant inverse associations between SHBG levels and risk of cholecystectomy, both in men and in women (Table 1). The associations in both sexes were attenuated but remained significant after further adjusting for lifestyle behaviors (model 2) and testosterone (model 3). The fully-adjusted HRs comparing the highest with the lowest quartiles of SHBG were 0.67 (95% CI: 0.59–0.77) in men (*P*-trend < 0.001) and 0.40 (95% CI: 0.36–0.43) in women (*P*-trend < 0.001). Each 1-SD increment in SHBG levels was associated with a 14% (95% CI: 9%-18%) lower risk of cholecystectomy in men and a 31% (95% CI: 28%-33%) lower risk in women. The association appeared to be nonlinear in women (*P*-nonlinearity < 0.0001) but not in men (*P*-nonlinearity = 0.58) (Fig. 1).

**Table 1:** Serum sex hormone-binding globulin (SHBG) and risk of cholecystectomy in men and women

	Sex-specific quartile for SHBG				*P*-trend	Per SD increment
	Q1	Q2	Q3	Q4		
**Men**						
Median (range), nmol/L	22.4 (< 27.7)	32.2 (27.7–36.6)	41.5 (36.7–47.6)	56.9 (≥ 47.7)		
No. of participants	44,195	44,252	44,207	44,255		
No. of cases/person-y	803/547,379	751/545,427	704/541,876	619/536,620		
Model 1 (HR [95% CI])	1.00 (referent)	0.86 (0.78–0.95)	0.76 (0.69–0.85)	0.64 (0.57–0.71)	< 0.001	0.85 (0.81–0.88)
Model 2 (HR [95% CI])	1.00 (referent)	0.86 (0.78–0.95)	0.77 (0.69–0.85)	0.63 (0.57–0.71)	< 0.001	0.85 (0.81–0.88)
Model 3 (HR [95% CI])	1.00 (referent)	0.88 (0.79–0.97)	0.80 (0.71–0.89)	0.67 (0.59–0.77)	< 0.001	0.86 (0.82–0.91)
**Women**						
Median (range), nmol/L	31.5 (< 40.5)	48.5 (40.5–56.7)	65.7 (56.8–77.7)	94.2 (≥ 76.8)		
No. of participants	40,017	40,047	40,037	40,046		
No. of cases/person-y	1745/491,357	1309/495,327	876/498,581	677/498,953		
Model 1 (HR [95% CI])	1.00 (referent)	0.75 (0.69–0.80)	0.49 (0.45–0.54)	0.38 (0.35–0.42)	< 0.001	0.68 (0.65–0.70)
Model 2 (HR [95% CI])	1.00 (referent)	0.78 (0.72–0.84)	0.52 (0.48–0.56)	0.39 (0.36–0.43)	< 0.001	0.69 (0.67–0.72)
Model 3 (HR [95% CI])	1.00 (referent)	0.78 (0.72–0.84)	0.52 (0.48–0.57)	0.40 (0.36–0.43)	< 0.001	0.69 (0.67–0.72)

Model 1: age (y), ethnic group (White, Asian/Asian British, Black/Black British, mixed), and Townsend deprivation index

Model 2: model 1 + smoking (never, former, current [< 10, 10-<50, ≥ 50 pack-years]), alcohol drinking (never, former, current < 10 g/d in men or < 5 g/d in women, current 10-<25 g/d in men or 5-<15 g/d in women, current ≥ 25 g/d in men or ≥ 15 g/d in women), total physical activity (MET-h/week), and for women, menopausal status (yes, no) and hormone replacement therapy (ever, never)

Model 3: model 2 + testosterone (nmol/L)

**Table 2:** Serum testosterone and risk of cholecystectomy in men and women

	Sex-specific quartile for testosterone				*P*-trend	Per SD increment
	Q1	Q2	Q3	Q4		
**Men**						
Median (range), nmol/L	8.08 (< 9.44)	10.5 (9.44–11.6)	12.7 (11.6–14.1)	16.0 (≥ 14.1)		
No. of participants	44,215	44,226	44,236	44,232		
No. of cases/person-y	842/539,332	767/543,266	678/544,777	590/543,926		
Model 1 (HR [95% CI])	1.00 (referent)	0.92 (0.83–1.01)	0.81 (0.73–0.90)	0.72 (0.65–0.80)	< 0.001	0.88 (0.85–0.92)
Model 2 (HR [95% CI])	1.00 (referent)	0.93 (0.84–1.03)	0.83 (0.75–0.92)	0.73 (0.66–0.81)	< 0.001	0.89 (0.86–0.92)
Model 3 (HR [95% CI])	1.00 (referent)	1.00 (0.90–1.11)	0.95 (0.85–1.06)	0.90 (0.80–1.03)	0.088	0.97 (0.92–1.01)
**Women**						
Median (range), nmol/L	0.56 (< 0.72)	0.87 (0.72–1.02)	1.17 (1.02–1.36)	1.67 (≥ 1.37)		
No. of participants	39,911	40,113	40,065	40,058		
No. of cases/person-y	1058/494,210	1126/497,376	1092/497,136	1331/495,496		
Model 1 (HR [95% CI])	1.00 (referent)	1.06 (0.98–1.15)	1.03 (0.95–1.13)	1.26 (1.16–1.37)	< 0.001	1.10 (1.07–1.13)
Model 2 (HR [95% CI])	1.00 (referent)	1.08 (0.99–1.18)	1.07 (0.98–1.16)	1.32 (1.21–1.43)	< 0.001	1.11 (1.08–1.14)
Model 3 (HR [95% CI])	1.00 (referent)	1.08 (0.99–1.17)	1.06 (0.97–1.15)	1.28 (1.18–1.38)	< 0.001	1.10 (1.07–1.13)

Model 1: age (y), ethnic group (White, Asian/Asian British, Black/Black British, mixed), and Townsend deprivation index

Model 2: model 1 + smoking (never, former, current [< 10, 10-<50, ≥ 50 pack-years]), alcohol drinking (never, former, current < 10 g/d in men or < 5 g/d in women, current 10-<25 g/d in men or 5-<15 g/d in women, current ≥ 25 g/d in men or ≥ 15 g/d in women), total physical activity (MET-h/week), and for women, menopausal status (yes, no) and hormone replacement therapy (ever, never)

Model 3: model 2 + SHBG (nmol/L)

### Testosterone and risk of cholecystectomy

After the adjustment for sociodemographic factors, testosterone was significantly and inversely associated with risk of cholecystectomy in men (*P*-trend < 0.001), but was positively associated with risk in women (*P*-trend < 0.001) (Table 2). In men, the association remained significant after further adjusting for lifestyle behaviors (model 2). However, after an additional adjustment for SHBG (model 3), there was no association between testosterone and risk of cholecystectomy in men (HR_Q4 vs. Q1_ = 0.90, 95% CI: 0.80–1.03; *P*-trend = 0.088).

In women, the positive association between testosterone and risk of cholecystectomy persisted in the fully adjusted model (model 3), with an HR of 1.28 (95% CI: 1.18–1.38; *P*-trend < 0.001) comparing the highest with the lowest quartiles of testosterone. Each 1-SD increment in testosterone levels was associated with a 10% (95% CI: 7%-13%) higher risk of cholecystectomy. There was no evidence for a nonlinear association between testosterone and cholecystectomy risk either in men or in women (Fig. 1).

### Subgroup and supplementary analyses

In men, the inverse association between SHBG levels and risk of cholecystectomy was broadly consistent across various subgroups defined by demographic characteristics and other risk factors (Supplementary Fig. 2). In women, there was evidence for a stronger association among those who were never or former smokers, never or current drinkers, or never users of HRT than the association compared with the corresponding subgroups. For testosterone, the association with cholecystectomy in men was inverse in non-white ethnicity and in former drinkers, whereas the association in women appeared to be stronger in current drinkers than in never or former drinkers without other group differences (Supplementary Fig. 3).

The observed associations between SHBG (Supplementary Fig. 4) or testosterone (Supplementary Fig. 5) and risk of cholecystectomy were similar after excluding the prevalent disorders which may affect hormone metabolism, excluding participants without cholelithiasis before the cholecystectomies, or adjusting for dietary and metabolic factors potentially influencing hormone levels. However, for both SHBG and testosterone, their associations with cholecystectomy were moderately attenuated after further adjusting for BMI or waist circumference, regardless of sex. Analyses using calculated cFT showed broadly similar patterns to those observed for total testosterone (Supplementary Table 2), with no clear association in men and a positive association in women.

### Mediation of testosterone and SHBG on obesity-cholecystectomy relationship

**Fig. 2:**
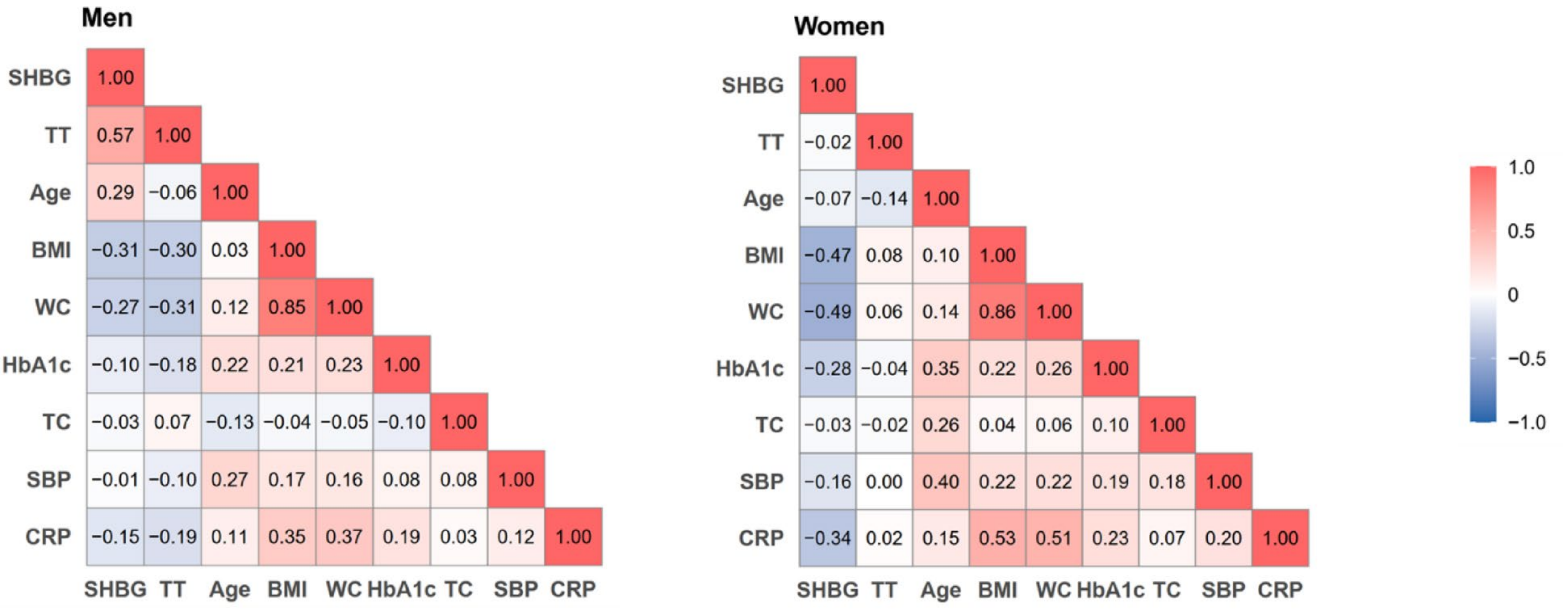
Spearman correlations between serum SHBG and testosterone and other risk factors. BMI, body mass index; CRP, C-reactive protein; HbA1c, glycosylated hemoglobin; SBP, systolic blood pressure; SHBG, sex hormone-binding globulin; TC, total cholesterol; TT, testosterone; WC, waist circumference

There was a moderate positive correlation between SHBG and testosterone in men (*r* = 0.57) but not in women (*r* = -0.02) (Fig. 2). SHBG was moderately and inversely correlated with BMI and waist circumference, more strongly in women than in men, whereas testosterone was inversely associated with BMI and waist circumference in men, but the correlations in women were weak and positive. In addition, SHBG was inversely correlated with HbA1c, SBP, and CRP more evidently in women than in men, whereas testosterone was inversely correlated with these traits only in men.

**Fig. 3:**
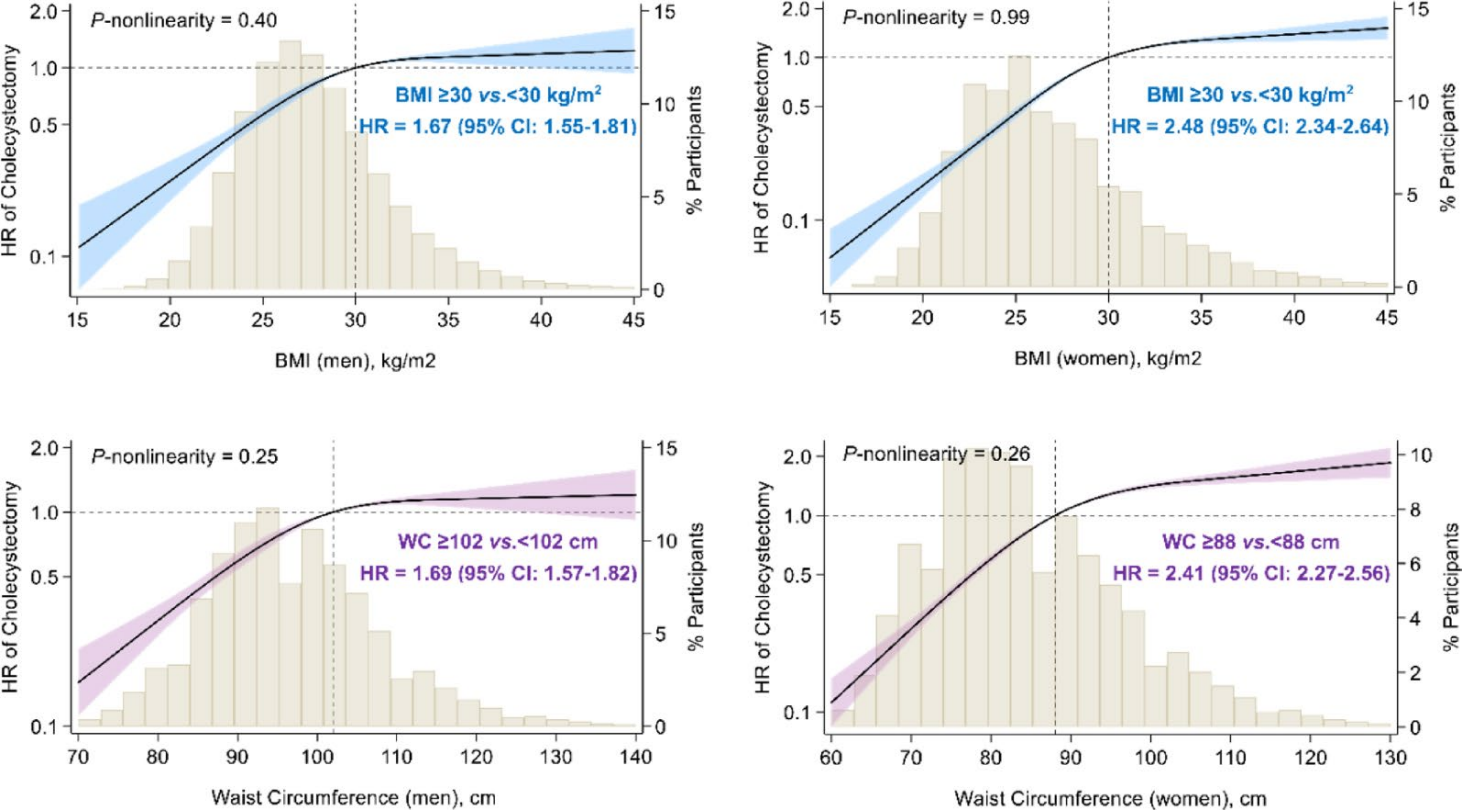
Dose-response relationships of general and abdominal adiposity measures with risk of cholecystectomy in men and women. BMI, body mass index; WC, waist circumference

After the multivariable adjustment, higher BMI and waist circumference were both associated with a higher risk of cholecystectomy in a dose-response manner, irrespective of sex (all *P*-nonlinearity values > 0.25) (Fig. 3). General obesity was associated with a 148% (HR = 2.48, 95% CI: 2.34–2.64) higher risk of cholecystectomy in women and a 67% (HR = 1.67, 95% CI: 1.55–1.81) higher risk in men (P for interaction < 0.001). Similarly, abdominal obesity was associated with a 141% (HR = 2.41, 95% CI: 2.27–2.56) higher risk of cholecystectomy in women and a 69% (HR = 1.69, 95% CI: 1.57–1.82) higher risk in men (P for interaction < 0.001).

**Fig. 4:**
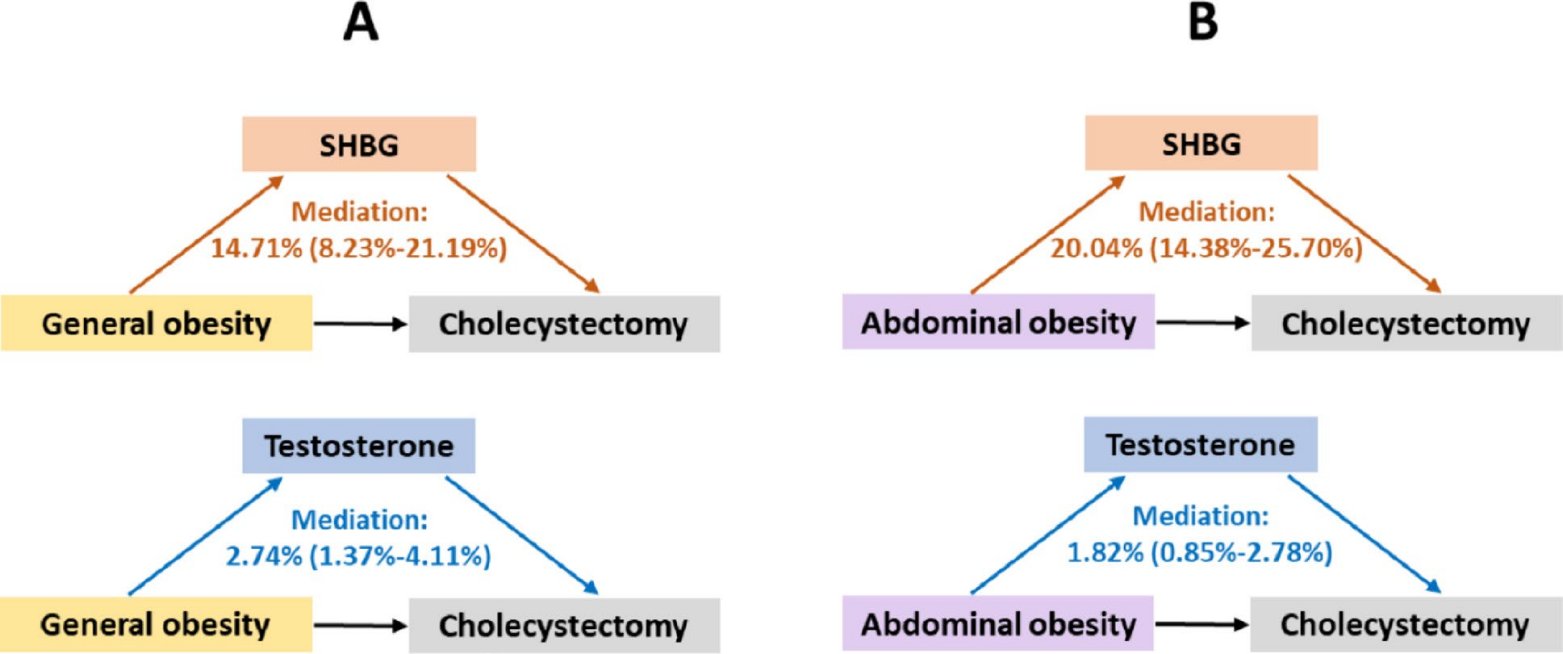
Mediation of SHBG and testosterone in the relationship between general obesity (**A**) or abdominal obesity (**B**) and risk of cholecystectomy in women. SHBG, sex hormone-binding globulin

For women, results from the mediation analysis indicated that SHBG and testosterone significantly mediated 14.7% (95% CI: 8.2%-21.2%) and 2.7% (95% CI: 1.4%-4.1%) of the association between general obesity and cholecystectomy, respectively (Fig. 4A). For the abdominal obesity-cholecystectomy association in women, the proportions of mediation by SHBG and testosterone were 20.0% (95% CI: 14.4%-25.7%) and 1.8% (95% CI: 0.9%-2.8%), respectively (Fig. 4B). No significant mediation effect was observed for either SHBG or testosterone in men (data not shown). 

## Discussion

In this prospective analysis of middle-aged and older adults in the UK, we systematically assessed the associations of serum SHBG and testosterone levels with risk of cholecystectomy in men and women. Higher levels of SHBG were associated with a lower risk of cholecystectomy in both sexes, whereas higher levels of testosterone were associated with a higher risk of cholecystectomy only in women. Obesity showed sex-specific associations with risk of cholecystectomy, with a substantially stronger association in women than in men. In women, statistical mediation analyses suggested that SHBG, and to a lesser extent testosterone, accounted for a modest proportion of the obesity-cholecystectomy association, whereas no significant mediation was observed in men.

Very few previous studies have assessed circulating levels of SHBG in relation to gallstone-related outcomes. A small Italian case-control study reported no significant difference in SHBG levels between gallstones cases and controls [38]. In a Danish cohort study, an inverse association between SHBG and incident gallstones was attenuated after adjustment for BMI but remained evident for longitudinal increases in SHBG [39]. In the present study with a substantially larger number of outcome events, we found a consistent inverse association in both sexes, with a stronger association in women than in men.

SHBG is synthesized predominantly by hepatocytes, which also play central roles in bile acid synthesis and hepatobiliary cholesterol secretion [40–42]. Experimental evidence indicates that hyperinsulinemia associated with insulin resistance suppresses hepatic SHBG expression, likely through inhibition of HNF-4α-mediated transcription [43]. Consistent with this mechanism, higher circulating SHBG levels have been associated with a lower risk of type 2 diabetes, reflecting better insulin sensitivity and a more favorable metabolic profile. Hepatic insulin resistance has been shown to alter cholesterol metabolism, including increased expression of biliary cholesterol transporters (ABCG5/ABCG8), enhanced cholesterol secretion into bile, and changes in bile acid synthetic pathways [44]. These changes may promote cholesterol supersaturation of bile, a key initiating step in gallstone formation. Accordingly, lower SHBG concentrations may reflect a hepatic metabolic state characterized by insulin resistance and lithogenic bile composition. Importantly, SHBG transports androgens and estrogens in the circulation and regulates their access to target tissues [22]. Changes in SHBG levels influence the plasma distribution of these hormones between bound and free fractions [22]. Therefore, circulating SHBG concentrations may reflect, at least in part, an integrated hormonal-metabolic state rather than exerting a direct causal effect. These mechanistic considerations provide a biological basis for our mediation findings, suggesting that part of the adiposity-related risk may operate through hepatic metabolic alterations reflected by SHBG levels.

With respect to testosterone, the observed association with cholecystectomy differed substantially by sex. In men, the inverse association observed in initial models was attenuated to be null after adjustment for SHBG, suggesting collinearity between these biomarkers or shared underlying metabolic pathways rather than an independent effect of testosterone. In women, higher testosterone levels remained independently associated with an increased cholecystectomy risk. Although the absolute differences in testosterone levels across quartiles were modest, the physiological range of circulating testosterone in women is relatively narrow and tightly regulated. In this context, even small elevations may be biologically meaningful. Prior evidence indicates that higher circulating testosterone levels in women are associated with adverse metabolic profiles, including insulin resistance and metabolic syndrome [45, 46]. These findings raise the possibility that elevated testosterone in women may reflect broader metabolic dysregulation rather than representing an isolated endocrine abnormality. Nevertheless, experimental studies provide biological plausibility for a direct role of testosterone in gallstone formation. Animal studies have demonstrated that administration of methyltestosterone markedly increases cholesterol gallstone formation in female hamsters under lithogenic dietary conditions [47]. At the cellular level, testosterone has been shown to inhibit cholecystokinin (CCK)-induced gallbladder smooth muscle contraction via rapid non-genomic mechanisms involving intracellular calcium signaling [48]. Such effects could potentially reduce gallbladder contractility under physiological conditions.

The sex-specific difference in the association between testosterone and cholecystectomy may involve the role of estrogen. The sex difference in gallstone prevalence is thought to be driven, in part, by endogenous estrogens, particularly higher estrogen exposure, which promotes cholesterol gallstone formation by increasing hepatic secretion of biliary cholesterol and cholesterol saturation of bile [49–51]. Testosterone, through aromatization, can be converted to estrogen [52], raising the possibility that alterations in the androgen-estrogen balance may contribute to gallstone risk, although this interpretation remains speculative because estrogens were not directly measured in the present study.

In line with previous studies, obesity was found to be associated with cholecystectomy risk in both sexes, with a substantially stronger effect in women [26]. Obesity is increasingly recognized as a neuroendocrine disorder involving complex neuroendocrine mechanisms affecting hypothalamic and reward circuits that regulate feeding behavior and energy balance [53]. In particular, orexin (hypocretin) neurons in the lateral hypothalamus integrate peripheral metabolic signals with the mesolimbic dopamine reward system, enhancing the rewarding value of palatable foods and promoting intake beyond energetic needs [54]. Experimental studies further show that palatable high-fat diets rapidly remodel synaptic inputs onto orexin neurons, supporting a neural basis for sustained overeating and weight gain [55, 56]. These central neurobehavioral alterations may contribute to maladaptive eating patterns, including binge-like behaviors. At the level of the gut-brain axis, binge-eating phenotypes have been associated with blunted postprandial CCK release [57], a key hormonal signal triggering gallbladder contraction and bile emptying [58]. Impaired CCK signaling could plausibly contribute to gallbladder dysmotility, bile stasis, and a more lithogenic biliary environment [59]. Although speculative, these central-peripheral interactions provide a plausible upstream framework linking obesity, hormonal alterations, and gallbladder disease risk.

### Strengths and limitations

To our knowledge, this is the first prospective study to systematically examine the sex-specific associations of serum levels of SHBG and testosterone with risk of cholecystectomy. The study utilized a large sample size with long-term follow-up and an adequate number of outcome events. We comprehensively incorporated a wide range of potential confounders into the multivariable models to enhance the robustness of the findings.

Some potential limitations should be acknowledged. First, gallstone formation reflects an underlying metabolic and biliary process, whereas symptomatic gallstone disease represents a clinical manifestation of this process. Cholecystectomy represents a clinical and therapeutic decision rather than a direct measure of gallstone occurrence. Surgical intervention may be influenced by symptom severity, healthcare utilization, access to care, and local clinical practice patterns. Accordingly, residual bias related to surgical decision-making cannot be fully excluded. Additionally, sex differences in symptom presentation, healthcare-seeking behavior, and surgical thresholds may also affect the likelihood of undergoing cholecystectomy [60]. The stronger associations observed in women should be interpreted with recognition that sex-specific differences in clinical decision-making may have partly contributed to these findings. Second, due to the observational nature of the study, causality cannot be established. Although we adjusted for a wide range of known confounders, residual confounding cannot be completely excluded. In addition, several lifestyle variables, including smoking, alcohol consumption, diet, and physical activity, were self-reported and therefore subject to recall and reporting bias, which may have introduced residual confounding. Third, BMI and circulating hormone concentrations were measured only once at baseline. Intra-individual variability in body weight and hormonal profiles over time, particularly age-related fluctuations and changes during midlife and the menopausal transition, may have resulted in exposure misclassification and regression dilution bias. Such non-differential misclassification would generally attenuate true associations toward the null. Because trajectories of adiposity and sex hormones differ between men and women, the magnitude of this bias may not be uniform across sexes and could partly influence the interpretation of sex-specific associations. In addition, sex hormone levels fluctuate across the menstrual cycle in premenopausal women. This may have introduced random measurement error among younger participants. However, given the broad age range and long follow-up, such variability is unlikely to have materially influenced the overall findings. Fourth, the assay used to measure estradiol was unable to reliably quantify concentrations below 175 pmol/L, leaving a substantial proportion of participants below the detection threshold [61]. Progesterone was not systematically measured. Consequently, estradiol and progesterone were not included in the analyses, limiting our ability to directly evaluate estrogen-mediated mechanisms. In addition, other physiological or exogenous hormonal states, such as pregnancy and oral contraceptive use, were not explicitly accounted for. Although these exposures were uncommon in this age group, they may influence circulating hormone levels and gallstone risk. Fifth, mediation analyses may be affected by complex confounding structures. Some potential mediator-outcome confounders, such as insulin resistance or metabolic inflammation, may themselves lie on the causal pathway from obesity to gallbladder disease. Therefore, the estimated mediation proportions should be interpreted cautiously as statistical approximations rather than definitive mechanistic quantification. Finally, the study population consisted predominantly of individuals of European ancestry who tend to be healthier and of higher socioeconomic status than the general UK population [62]. These characteristics may limit the generalizability of our findings to other ethnic and regional populations.

## Conclusions

In summary, our findings suggest that serum SHBG levels are inversely associated with risk of cholecystectomy irrespective of sex, whereas testosterone levels are positively associated with risk of cholecystectomy only in women. Obesity exhibits a stronger association with cholecystectomy in women than in men. SHBG and, to a lesser extent, testosterone may account for a modest proportion of the obesity-cholecystectomy association in women. Further mechanistic and experimental studies are needed to clarify the underlying biological pathways.

## Supplementary Information

Below is the link to the electronic supplementary material.

Supplementary Material 1.

## Data Availability

UK Biobank data can be requested by bona fide researchers for approved projects, including replication, through [https://www.ukbiobank.ac.uk/](https:/www.ukbiobank.ac.uk) .

## Abbreviations

BMI: Body mass index
CI: Confidence interval
HbA1c: Hemoglobin A1c
HR: Hazard ratio
HRT: Hormone replacement therapy
MET: Metabolic equivalent
SD: Standard deviation
SHBG: Sex hormone-binding globulin
